# Discovery of N-(2-Amino-4-Fluorophenyl)-4-[*bis*-(2-Chloroethyl)-Amino]-Benzamide as a Potent HDAC3 Inhibitor

**DOI:** 10.3389/fonc.2020.592385

**Published:** 2020-10-15

**Authors:** Yiming Chen, Jinhong Feng, Yajie Hu, Xuejian Wang, Weiguo Song, Lei Zhang

**Affiliations:** ^1^Department of Medicinal Chemistry, School of Pharmacy, Weifang Medical University, Weifang, China; ^2^Key Laboratory for Applied Technology of Sophisticated Analytical Instruments of Shandong Province, Analysis and Test Center, Qilu University of Technology (Shandong Academy of Sciences), Jinan, China

**Keywords:** 4-fluorine-benzamide, nitrogen mustard, HDAC, antitumor activity, isoform selectivity

## Abstract

In discovery of HDAC inhibitors with improved activity and selectivity, fluorine substitution was performed on our previously derived lead compound. The synthesized molecules N-(2-amino-4-fluorophenyl)-4-[*bis*-(2-chloroethyl)-amino]-benzamide (FNA) exhibited class I (HDAC1, 2, and 3) selectivity in the *in vitro* enzymatic assay and especially potent against HDAC3 activity (IC_50_: 95.48 nM). The results of *in vitro* antiproliferative assay indicated that FNA exhibited solid tumor cell inhibitory activities with IC_50_ value of 1.30 μM against HepG2 cells compared with SAHA (17.25 μM). Moreover, the *in vivo* xenograft model study revealed that FNA could inhibit tumor growth with tumor growth inhibition (TGI) of 48.89% compared with SAHA (TGI of 48.13%). Further HepG2 cell–based apoptosis and cell cycle studies showed that promotion of apoptosis and G2/M phase arrest make contributions to the antitumor activity of FNA. In addition, drug combination results showed that 0.5 μM of FNA could improve the anticancer activity of taxol and camptothecin. The present studies revealed the potential of FNA utilized as a high potent lead compound for further discovery of isoform selective HDAC inhibitors.

## Introduction

Histone deacetylases and histone acetylases are important enzymes participating in the regulation of gene expression by acetylating and deacetylating of histones (1, 2). Specifically, HDACs are the enzymes controlling the epigenetic modifications of histone, along with more than 50 nonhistone proteins (3, 4). So far, a total of 18 different HDACs isoforms have been identified and classified into four classes according to their size, distribution in cells, and homology (5–8). Among the four classes, classes I (HDAC1, 2, 3, and 8), II (HDAC4, 5, 6, 7, 9, and 10), and IV (HDAC11) HDACs require zinc ion as cofactor and thus are known as zinc-dependent enzymes. On the other hand, class III HDACs are a group of NAD+-dependent enzymes (also known as sirtuins), whose activity does not require the presence of zinc iron (9–13).

In recent years, inhibition of HDACs has exhibited potency for the treatment tumors (14, 15), diabetes (16), Parkinson disease (17), inflammation (18, 19), HIV (20), and heart disease (21). In tumor cells, it had been shown that overexpression of HDACs led to increased deacetylation of histones, which increases the gravitational pull between DNA and histones by restoring the positive charge of the histones, making the relaxed nucleosomes very tight and unfavorable for the expression of specific genes, including some tumor suppressor genes (22–28).

In the field of epigenetics, HDAC inhibitors (HDACIs) have been successfully developed in the antitumor therapy, and several HDACIs have been developed into the market (29). Vorinostat (SAHA) is the first approved HDACI, which has been administered clinically for the treatment of cutaneous T-cell lymphoma (CTCL) (30). Afterward, romidepsin (FK-228), belinostat (PXD101), and panobinostat (LBH589) were approved for the treatment of CTCL, peripheral T-cell lymphoma (PTCL), and multiple myeloma, respectively (31–33). Chidamide (CS055) was approved by the Chinese Food and Drug Administration for the treatment of PTCL (34). Generally, pharmacophores of HDACIs are consist of three structural elements: a capping group, which recognizes the hydrophobic region at the opening of HDAC active site; a linker, which connects the hydrophobic ring and the zinc-binding group (ZBG) via occupation of the tubular channel; a ZBG, whose functions include binding to the zinc ion located in the active center of HDACs, as well as forming hydrogen bonds with certain amino acid residues of active sites (35–37).

Nitrogen mustard anticancer drugs were used clinically since 1942, which effectively bind and cross-link to DNA, resulting in prevention of DNA replication and cell proliferation (38). Nitrogen mustard antitumor drugs are mainly composed of alkylation part and carrier part. According to different carriers, they can be divided into aliphatic nitrogen mustard and aromatic nitrogen mustard (39). Aromatic nitrogen mustard is still used in clinical because of its relatively low toxicity such as chlormethine (40), chlorambucil (41), and melphalan (42).

**Figure 1:**
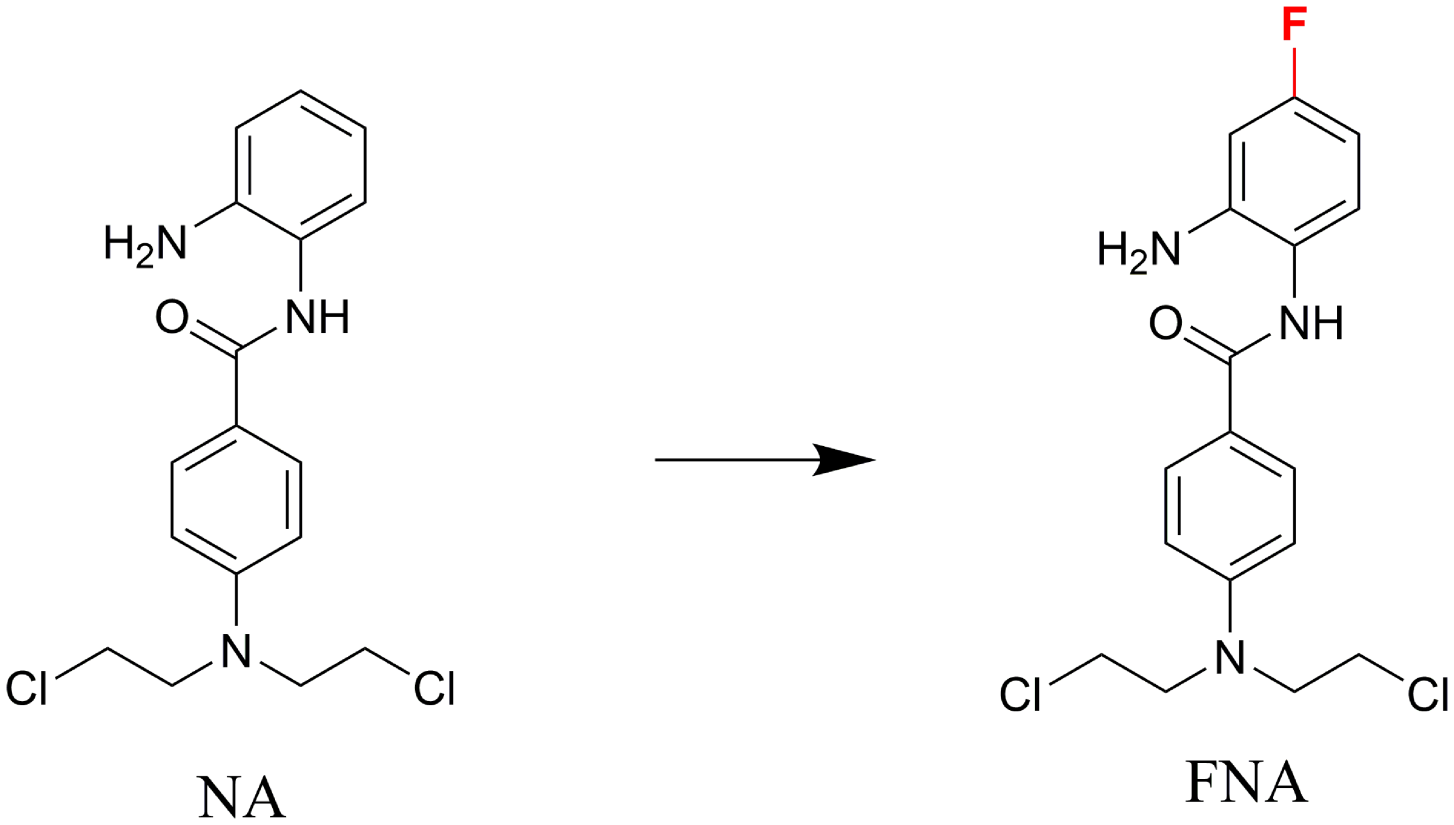
The design of FNA.

In discovery of novel and potent HDACIs, aromatic nitrogen mustard parts were integrated into the structure of HDACI CI994 in our previous study (43). The resulting molecule, N-(2-aminophenyl)-4-(*bis*(2-chloroethyl)amino)benzamide (NA) exhibited class I selectivity in the enzymatic assay and potent *in vitro* antitumor activity in the cell based assay. However, NA exhibited lower potency than SAHA in the *in vivo* assay using nude mice xenograft model with inoculation of HepG2 cells. Fluorine substitution in the benzamide ZBG was discovered to improve the metabolic stability of HDACIs, such as the design of chidamide (44, 45). In the present study, to improve the selectivity, activity, and *in vivo* stability of NA, fluorine was introduced to the *para*-position of amide bond in the phenyl ring of ZBG considering the structure of chidamide (Figure 1). The designed compound N-(2-amino-4-fluorophenyl)-4-[*bis*-(2-chloroethyl)-amino]-benzamide (FNA) was synthesized and evaluated in the antitumor assay.

## Results and Discussion

### Chemistry

**Scheme 1:**
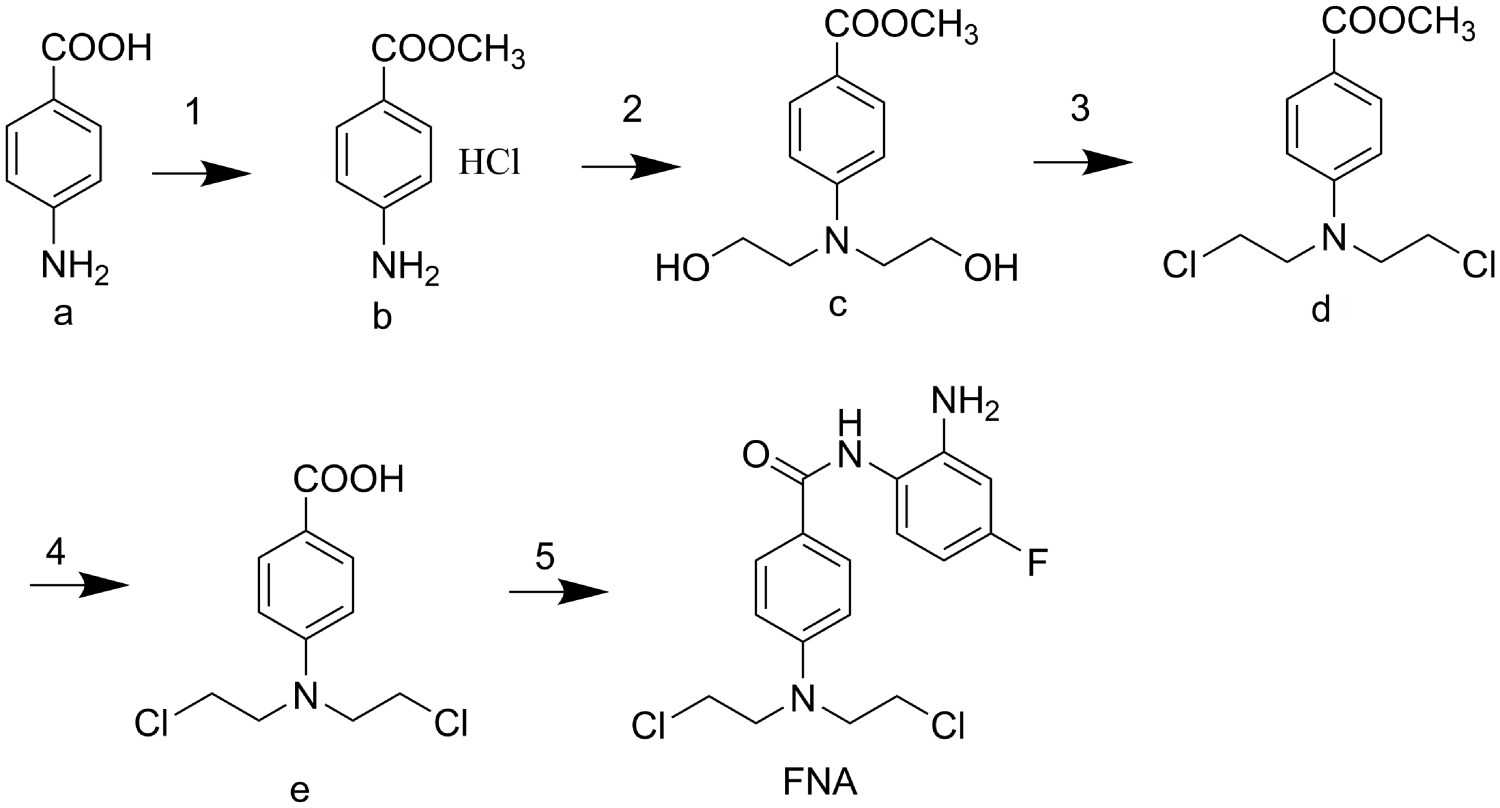
(1) CH_3_COCl, MeOH, reflux 5 h. (2) oxirane, H_2_O/CH_3_COOH, 0°C 48 h. (3) POCl_3_, MB, reflux 4 h. (4) 4 M HCl, reflux 4 h. (5) 1,2-diamino-4-fluorobenzene, CDI, THF (dry), rt overnight.

The designed compound FNA was synthesized as described in Scheme 1. Methyl esterification was performed to protect the starting material 4-aminobenzoic acid (a). To synthesize intermediate methyl 4-(*bis*(2-hydroxyethyl)amino)benzoate (c), 2-hydroxyethyl groups were added by reaction of intermediate **b** with ethylene oxide. Subsequent chlorine substitution and deprotection of carboxyl group afforded key intermediate 4-(*bis*(2-chloroethyl)amino)benzoic acid (e). Target compound FNA was synthesized by condensation of intermediate **e** with 1,2-diamino-4-fluorobenzene.

### Enzyme Inhibitory Selectivity of FNA

To assess the isoform selectivity and the inhibitory activity of the derived FNA, enzymatic assay was performed against HDAC1, 2, 3, 4, 6, 7, 8, and 9 using SAHA (nonselective inhibitor) and MS275 (class I selective inhibitor) as positive control drugs. The selectivity of isoforms and IC_50_ of the tested compounds were displayed in Table 1. According to the results, FNA exhibited IC_50_ values of 842.80, 949.15, and 95.48 nM against HDAC1, 2, and 3, respectively. While in inhibition of HDAC4, 6, 7, 8, and 9, FNA exhibited more than 5,000 nM of IC_50_ values. It is suggested that FNA is a highly class I–selective inhibitor. Nevertheless, in the inhibition of HDAC1, 2, and 3, it is remarkable that FNA showed 8.83- and 9.94-fold of HDAC3 selectivity vs. HDAC1 and HDAC2, respectively. The results suggested that FNA has the potential to be utilized as a lead compound for the discovery of HDAC3-selective inhibitors.

**Table 1:** Enzyme inhibitory activity of FNA compared with MS275 and SAHA (IC_50_, nM)^a^.

**HDACs**	**HDAC1**	**HDAC2**	**HDAC3**	**HDAC4**	**HDAC6**	**HDAC7**	**HDAC8**	**HDAC9**
FNA	842.80	949.15	95.48	>5,000	>5,000	>5,000	>5,000	>5,000
MS275	46.17	100.90	43.89	>5,000	>5,000	>5,000	>5,000	>5,000
SAHA	52.90	90.78	167.24	>5,000	172.10	>5,000	4,120	>5,000

a *Assays were performed in replicate (n ≥ 2), the SD values are <10% of the mean*.

Among all the HDAC isoforms found in human, HDAC3 is unique for its expression in the nucleus, cytoplasm, or membrane. As a single HDAC isoform, HDAC3 was revealed to promote the phosphorylation and activation of AKT, which specifically binds to HDAC3, participate in the self-renewal of liver cancer stem cells, and engage in the growth of triple-negative breast cancer cells (15). Therefore, discovery of selective HDAC3 inhibitors make contributions to the treatment of specific diseases related to the abnormal function of HDAC3. As a potent lead compound, FNA could be utilized for further structural modification in discovery of HDAC3-selective inhibitors.

### Antiproliferative Activity of FNA

The *in vitro* antiproliferative activities of target compound FNA along with the positive control SAHA were tested against multiple tumor cell lines, including the lung cancer (H460, H322, and A549), colon carcinoma SW480, renal carcinoma (OS-RC-2, SK-NEP-1), thyroid cancer (FTC-133, SW-579), breast cancer (MDA-MB-231), ovarian cancer (A2780), cervical carcinoma (Hela), myeloma (U266), liver cancer (HepG2), and leukemic (U937 and K562) cells. According to the results shown in Table 2, potent antiproliferative activities against most of the tumor cell lines tested (except SW480 and OR-RC-2) were observed from FNA, as evidenced by the low IC_50_ values. Compared with SAHA, it is obvious that FNA could effectively inhibit the growth of HepG2, U937, H460, FTC-133, HELA, and K562 cells with IC_50_ values of 1.30, 0.55, 4.73, 9.09, 1.41, and 1.31 μM, respectively. It showed that FNA has a significant inhibitory effect on both solid tumor cells and nonsolid tumor cells. Remarkably, in inhibition the growth of HepG2 cells, FNA (similar to NA) was revealed to be 13.3-fold (IC_50_ value of 1.30 μM) more potent relative to SAHA, whose IC_50_ value is 17.25 μM. The present results indicate the future of development of FNA analogs for the treatment of liver cancer.

**Table 2:** Antiproliferative activities of compound FNA against human cancer cells (IC_50_, μM)^a^.

**Cell line**	**FNA (μM)**	**SAHA (μM)**
HepG2	1.30 ± 0.25	17.25 ± 0.46
U937	0.55 ± 0.03	0.86 ± 0.03
H460	4.73 ± 0.05	7.63 ± 0.03
SW480	>100	2.91 ± 0.04
OS-RC-2	>100	>100
H322	6.36 ± 0.07	2.54 ± 0.06
SK-NEP-1	8.32 ± 0.18	3.68 ± 0.02
FTC-133	9.09 ± 0.13	24.30 ± 0.10
SW579	52.40 ± 0.13	24.30 ± 0.07
MDA-MB-231	35.29 ± 0.03	5.82 ± 0.08
A549	33.74 ± 0.04	4.92 ± 0.04
A2780	4.30 ± 0.12	2.71 ± 0.09
Hela	1.41 ± 0.04	1.89 ± 0.05
K562	1.31 ± 0.05	2.52 ± 0.05
U266	0.63 ± 0.32	0.22 ± 0.03

a *Assays were performed in replicate (n ≥ 2)*.

### *In vivo* Antitumor Activity

**Figure 2:**
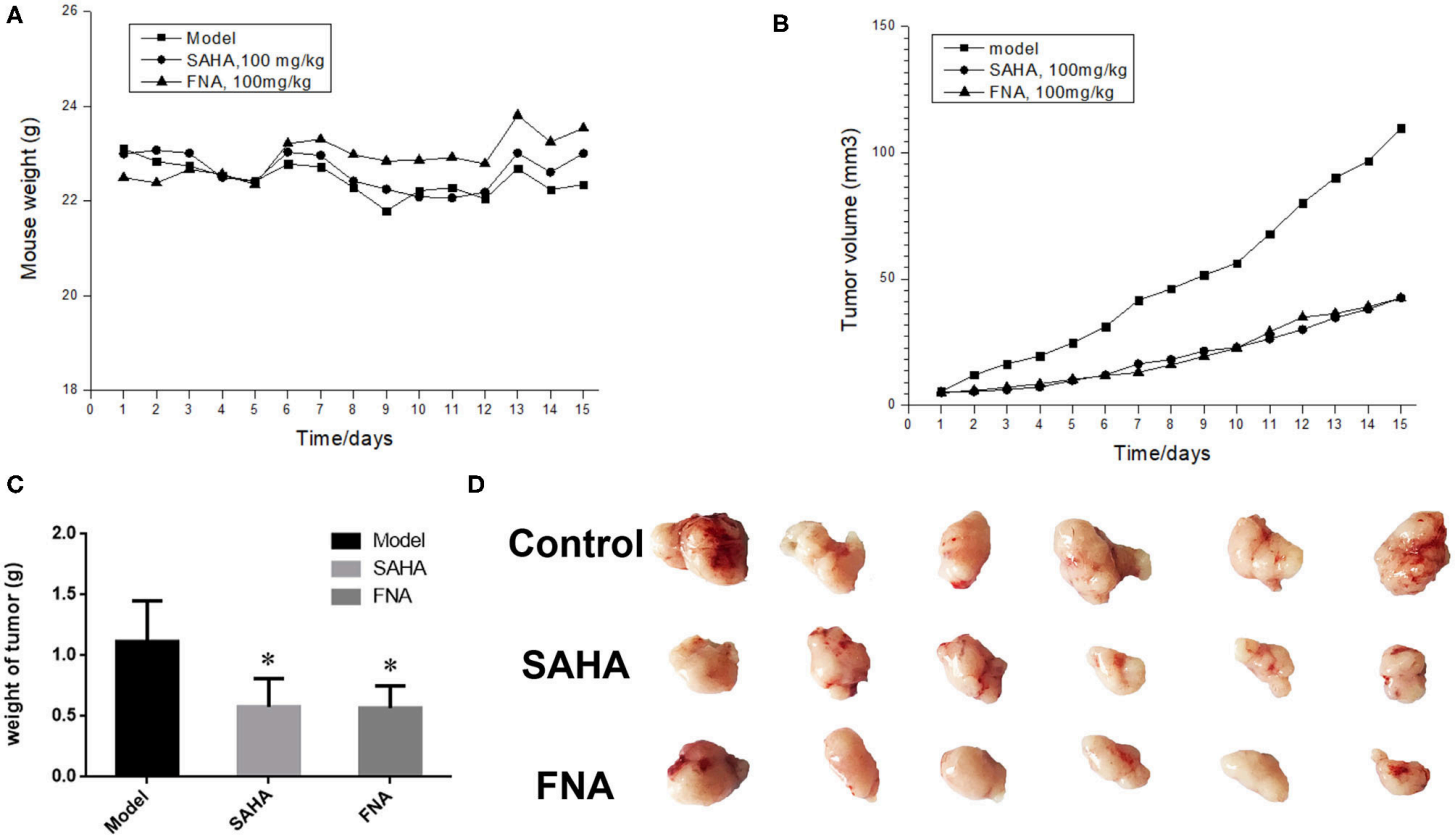
Results of *in vivo* evaluation of FNA in the HepG2 xenografts mouse model. **(A)** Body weight of mice. **(B)** Tumor volume measurements from HepG2 xenografts mice in each group. **(C)** The final tumor weights in each group. ^*^p < 0.05 compared with the control group. **(D)** Images of the excised tumors in each group.

To further investigate the anticancer activity of FNA, HepG2 xenograft nude mice model was utilized to assess the *in vivo* antitumor activity of compound FNA. Mice were injected intraperitoneally with FNA and SAHA, both at 100 mg/kg, once a day for 15 days. When the tumor is prominent, the BALB/c female mice were randomly assigned into control and treatment groups (six mice per group). As shown in Figure 2A, all the mice in the treatment groups displayed no significant change in body weight. The results showed that both FNA and SAHA can inhibit tumor growth compared with the control group (Figures 2B–D). Compound FNA effectively inhibited the tumor growth with tumor growth inhibition (TGI) rate of 48.89% compared with SAHA (with TGI of 48.13%). Although FNA exhibited improved inhibitory activity compared with SAHA in the *in vitro* test, the activity improvement was not obvious in the *in vivo* study. It is suggested that further structural modification of FNA is needed to improve the activity and pharmacokinetic properties. To improve the *in vivo* activity of FNA, introduction of bioactive groups with anticancer activities to the structure of FNA will be performed in further studies.

### Cell Apoptosis Analysis

**Figure 3:**
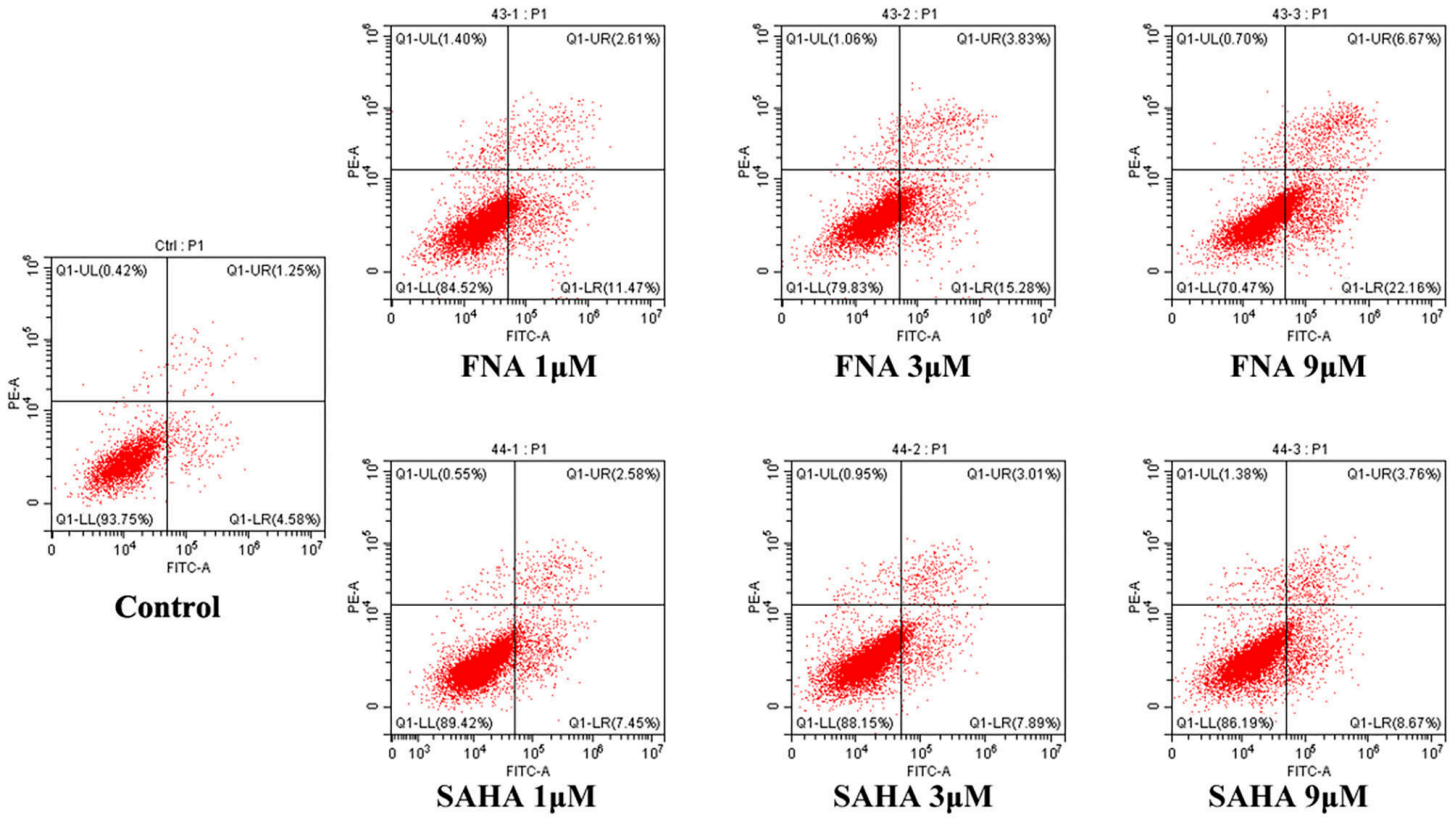
Proapoptotic effect of molecule FNA. HepG2 cells were exposed to FNA and SAHA at concentrations of 1, 3, and 9 μM for 24 h. Then cells were stained with annexin V–FITC/PI, and the apoptotic status of the cells was assessed with flow cytometry analysis.

In order to confirm whether apoptosis contributes to the observed antiproliferative activities of FNA, apoptosis study was performed using HepG2 cells. Flow cytometry analysis was shown in Figure 3. From the annexin V–fluorescein isothiocyanate/propidium iodide (FITC/PI) stating data, it is obvious that compound FNA promoted cell apoptosis against HepG2 cells dose-dependently. Following treatment with different doses of FNA (1, 3, and 9 μM), the apoptosis rate of HepG2 cells was significantly elevated from 5.83% of the normal group to 14.08, 19.11, and 28.83% compared with SAHA (apoptosis rate of 10.03, 10.91, and 12.43% at concentrations of 1, 3, and 9 μM), respectively. It is suggested that apoptosis plays a role in the HepG2 cell inhibitory activity of FNA.

### Cell Cycle Analysis

**Figure 4:**
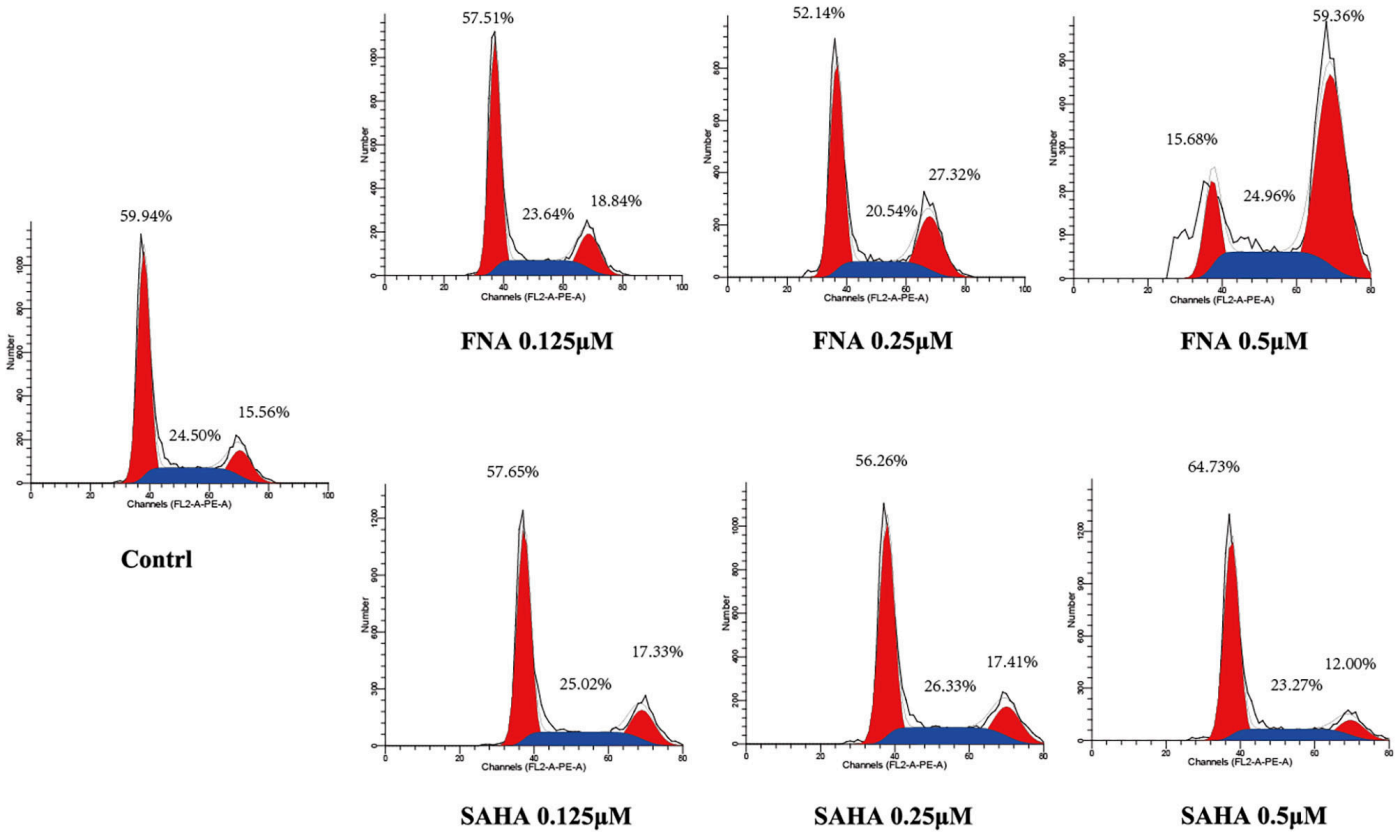
Cell cycle analysis on HepG2 cells treated with FNA. Cells were treated with FNA and SAHA at concentrations of 0.125, 0.25, and 0.5 μM for 6 h. The results were evaluated with flow cytometry analysis.

Generally, the cell cycle consists of three phases: G0/G1, S, and G2/M phase. A characteristic change in tumor cells is dysregulated cell cycle due to genetic mutations, resulting in uncontrolled cell proliferation. The designed compound FNA was evaluated for the cell cycle effect on HepG2 with various doses (0.125, 0.25, and 0.5 μM). As shown in Figure 4, it is significant that FNA increased cell number at G2/M phase with raising concentrations. The percentage of cells in G2/M phase was increased from 18.84 to 59.36% in the FNA (with concentration increase from 0.125 to 0.5 μM) group. However, at the tested concentrations, SAHA did not exhibit any effects in the regulation of HepG2 cell cycle. The results indicated that induction of the G2/M phase arrest also plays a significant role in the antiproliferative effects of molecule FNA.

### Antiproliferative Activities of FNA in Combination With Taxol and Camptothecin

**Figure 5:**
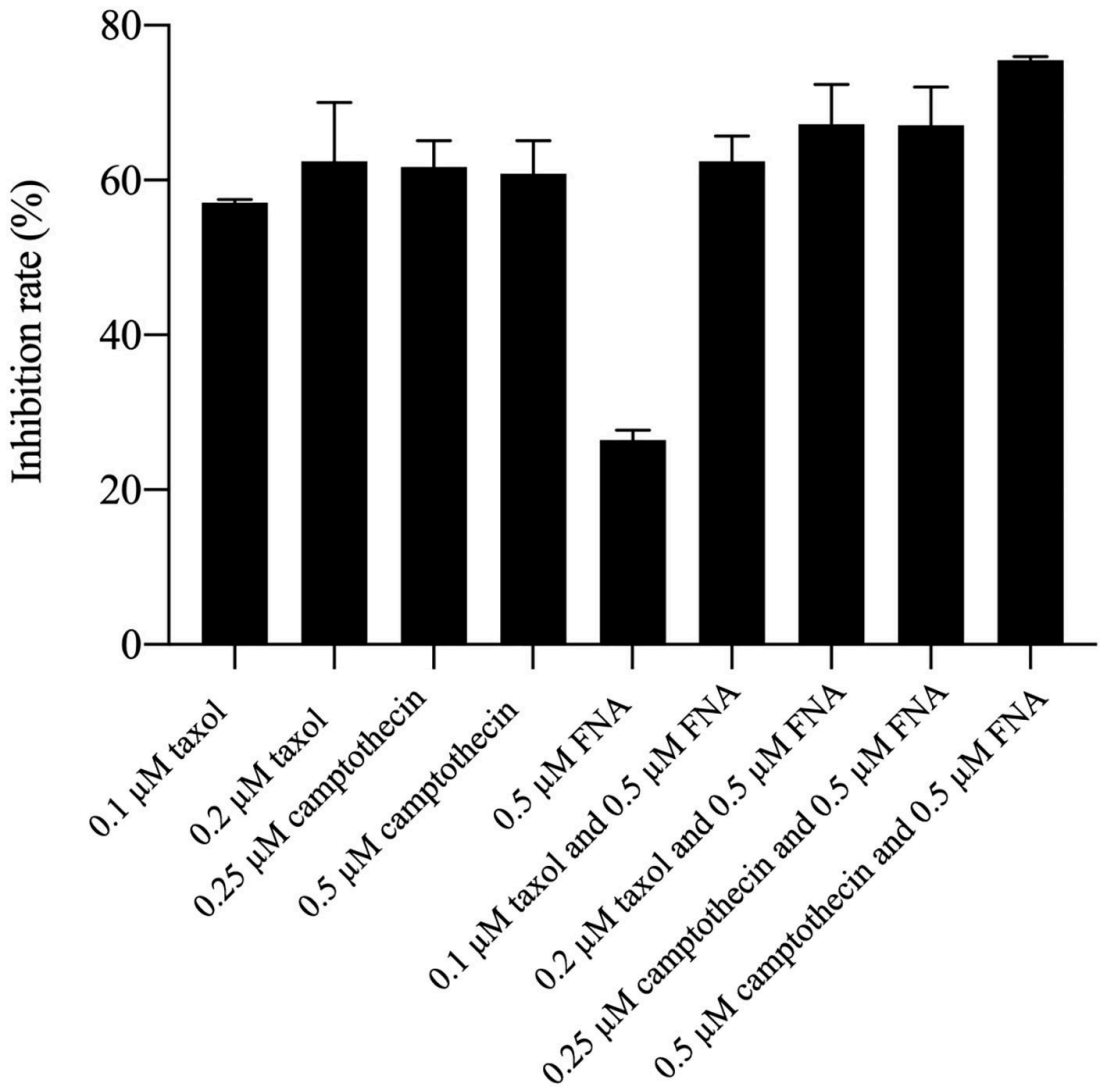
Antiproliferative activities of taxol and camptothecin alone and in combination with FNA.

It had been reported that HDACIs (HDACIs) may work as chemosensitizers when used together with other antitumor drugs (8). Because of the high cell cycle arrest ability of FNA, drug combination investigation was performed by combining FNA with the G2/M phase arrest drug taxol and camptothecin. HepG2 cells were used for the test, and percentage inhibition rate (PIR) was used as a measure of potency. As shown in Figure 5, it is revealed that the PIRs of FNA (0.5 μM) in combination with taxol and camptothecin are higher than that of the single-drug groups on HepG2 cells. The PIRs of taxol were 57.07 and 62.41 at the concentrations of 0.1 and 0.2 μM, respectively. Addition of 0.5 μM of FNA increased the PIR to 62.43 (0.1 μM of taxol) and 67.23 (0.2 μM of taxol), respectively. The PIRs of camptothecin at doses of 0.25 and 0.5 μM were 61.70 and 60.86, respectively. Improved activities were obtained by addition of FNA (0.5 μM) with PIR values of 67.07 (0.25 μM of camptothecin) and 75.52 (0.5 μM of camptothecin), respectively. It is suggested that FNA could synergistically improve the antiproliferative ability of the cell cycle arrest drugs such as taxol and camptothecin.

## Conclusion

Benzamide HDACIs exhibited the advantage of class I selectivity compared with HDACIs with hydroxamic acid as their ZBGs. However, none of the benzamide HDACIs have been approved by US Food and Drug Administration (FDA) yet. The fluorine substituted benzamide HDACI, chidamide, approved by the CFDA, exhibited advantage of high pharmacokinetic properties compared with the unsubstituted benzamide (such as MS275). Therefore, fluorine substituted was performed on the previous lead compound NA. The derived FNA exhibited HDAC3 selectivity and high HepG2 cell inhibitory activity. Moreover, FNA was also effective in the HepG2 nude mice xenograft model–based assay. Further investigations revealed that promotion of apoptosis and cell cycle arrest at G2/M phase both contributed to the antitumor activity of FNA. Moreover, in combination with FNA, taxol and camptothecin exhibited improved antiproliferative activities against HepG2 cells. Collectively, a potent HDAC3 inhibitor was discovered, which could be utilized as a lead compound in the development of new drugs for cancer treatments.

## Materials and Methods

### Chemistry

All the starting materials and reagents commercially available were used in the current study without further purifications. The dry THF was used by heating reflux with sodium. TLC with 0.25-mm silica gel plates (60GF-254) was used to monitor all the reactions. The sports were visualized with UV light and ferric chloride. With a Burker DRX spectrometer, the ^1^H NMR spectra were recorded at 500 MHz, using TMS as an internal standard. High-resolution mass spectra were performed at Weifang Medical University in Weifang, China. The derived target compound (FNA) is of 95.48% purity analyzed by ultraperformance liquid chromatography (UPLC), which was performed on a Waters Acquity H class UPLC instrument using an Acquity UPLC^®^BEH C18 (150 × 2.1 mm). The mobile phase was acetonitrile–water (90:10), and detection wavelength was 254 nm.

The synthesis and description of 4-(*bis*(2-chloroethyl)amino)benzoic acid (e) were presented in our previous work (43).

#### (2-Aminophenyl)-4-(*bis*(2-Chloroethyl)Amino)Benzamide

To a solution of compound e (2.00 g, 7.7 mmol) in THF (50 mL), CDI (1.87 g, 11.6 mmol) was added, and the solution was refluxed for 3 h. 1,2-Diamino-4-fluorobenzene (3.8 g, 30.6 mmol) and TFA (1.1 g, 9.24 mmol) were added with stirring, and the mixture was kept for 16 h at room temperature. The solvent was then evaporated with the residue being dissolved in EtOAc (50 mL). The resulting EtOAc solution was washed with NaHCO_3_ (3 × 20 mL), 1 M citric acid (3 × 20 mL), and brine (3 × 20 mL), dried over MgSO_4_, and evaporated under vacuum. The desired compound FNA was obtained by crystallization in EtOAc under 4°C as brown powder. HRMS (AP-ESI) m/z calculated for C_17_H_19_Cl_2_FN_3_O [M+H]^+^ 370.0889 found 370.0870. ^1^H NMR (400 MHz, (CD3)2SO): δ = 9.33 (s, ^1^H), 7.86 (d, *J* = 8.8 Hz, ^2^H), 7.08 (dd, *J*_1_ = 2.3 Hz, *J*_2_ = 6.4 Hz, ^1^H), 6.83 (d, *J* = 8.8 Hz, ^2^H), 6.54 (dd, *J*_1_ = 2.6 Hz, *J*_2_ = 11.2 Hz, ^1^H), 6.35 (td, *J*_1_ = 2.8 Hz, *J*_2_ = 8.5 Hz, ^1^H), and 5.14 (s, ^2^H), 3.86–3.75 (m, 8H). ^13^C NMR (400 MHz, (CD3)2SO): δ = 165.60, 161.32, 149.41, 145.81, 130.06, 127.80, 122.59, 120.42, 111.39, 102.60, 102.09, 52.33, and 41.52 ppm.

### Enzyme Inhibitory Selectivity of FNA

All of the HDAC enzymes tested were purchased from BPS Bioscience. First, 20 μL of each recombinant HDAC enzyme solution (HDAC1, 2, 3, 4, 6, 7, 8, and 9) was mixed with various concentrations of tested compound samples (20 μL) in a 96-well plate. The mixture was incubated at 30°C for 1 h for the dose-dependent assay. Additionally, mixtures were incubated for 15, 30, 60, and 90 min, for the time-dependent assay, and then 10 μL of fluorogenic substrate [3 mM Boc-Lys(acetyl)-AMC or Boc-Lys (trifluoroacetyl)-AMC for HDAC1/2/3/6 or HDAC4/7/8/9, respectively] was added. Then, the acetylation reaction was initiated by adding HDAC substrate working solution and incubating at 30°C for 2 h. After the desired time, 10 μL developer with trypsin and trichostatin A was added to stop the reaction, and then the mixture was incubated at 30°C for another 30 min.

A microplate reader was used to determine fluorescence intensity at excitation: 360 nm and emission: 460 nm. The inhibition ratios were calculated by comparing the fluorescence intensities from tested wells to those of controls. The IC_50_ curves and values were then obtained with GraphPad Prism 6.0 software.

### Antiproliferative Activity of FNA

Antiproliferative activities of FNA were evaluated with cell viability assay (MTT assay) with SAHA as the control drug (46). The stock solutions of compounds to be tested were prepared in culture medium. Tumor cell lines were cultured in 96-well plates at a density of 5 × 10^3^ cells per well and incubated until 90–95% confluence, and then each well received 100 μL medium containing desired concentrations of test compounds, and then incubated at 37°C and 5% CO_2_ for 48 h. To determine cell viability, 20 μL MTT working solution (5 mg/mL) was then added to each well and incubated for another 4 h. After this incubation, the medium was carefully aspirated, and 200 μL dimethyl sulfoxide (DMSO) was added to each well and vibrated for 10 min to make sure formed formazans were completely dissolved. The optical densities (ODs) at 490 and 630 nm were counted by Universal Microplate Spectrophotometer. The cell growth inhibition rate was calculated with the following equation: % inhibition = [1–(sample group OD_490_-sample group OD_630_)/(control group OD_490_-control group OD_630_)] × 100%. Origin 7.5 software was used to calculate the IC_50_ values from at least three independent experiments.

### *In vivo* Antitumor Activity

All animal experiments were performed in compliance with the Animal Experiment Ethical Review Board of Weifang Medical University. For *in vivo* antitumor efficacy studies, male athymic nude mice (5–6 weeks old, Slac Laboratory Animals, Shanghai, China) were inoculated subcutaneously in the right shoulder with 1.8 × 10^7^ HepG2 cells.

Injected mice were kept for 10 days; those with palpable tumors were then randomly assigned into treatment and control groups (six mice per group). The treatment groups were administrated with 100 mg/kg/d test compound intragastrically, whereas the control group was administered with an equal volume of phosphate-buffered saline (PBS) solution. The tumor size and body weight were assessed every day. After 15 days of treatment, the mice were euthanized, and tumor weights were acquired with an electronic balance. The TGI was calculated using the following formula: TGI = 100% × [1 – (TV_t(T)_ – TV_initial(T)_)/(TV_t(C)_ – TV_initial(C)_)], where TV_t(T)_ and TV_initial(T)_ stand for the mean tumor volume measured at final time and at initial time for the treatment groups, respectively, and TV_t(C)_ and TV_initial(C)_ represent the mean tumor volume for the control group.

### Cell Apoptosis Assay

HepG2 cells in logarithmic growth phase were cultured in 6-well plates (4 × 10^5^ cells per well).Various doses of FNA and SAHA (1, 3, and 9 μM) were added and incubated for 24 h. Then cells were washed with PBS, collected, and resuspended with binding buffer from a commercially available annexin V–FITC kit (Thermo Fisher Co., USA) and mixed with 5 μL of annexin V–FITC gently. Following 10 min of incubation, 1 μL of propidium iodide was added to the samples and incubated for another 20 min while avoiding light. Flow cytometry was used to determine cell apoptosis status (CytoFLEX, Beckman Coulter).

### Cell Cycle Analysis

HepG2 cells in logarithmic growth phase were cultured in 6-well plates with 6 × 10^5^ cells per well and incubated with different doses of FNA and SAHA (0.125, 0.25, and 0.5 μM). Following 6-h incubation, cells were washed twice with cold PBS and then fixed in 70% precooled ethanol at 4°C for 12 h. The fixed cells were washed again and then stained with PI/RNase A for 30 min at room temperature.

### Drug Combination Analysis

The efficacy of drug combination of FNA with taxol and camptothecin was evaluated via cell viability assay (MTT assay). The stock solutions of tested compounds were diluted to the desired concentrations with culture medium. The cells were cultured in 96-well plates at a density 5 × 10^3^ cells per well and incubated until 90–95% confluence, and then 100 μL of medium containing desired concentrations of test compounds was added to the wells. Following 48-h incubation, 10 μL of MTT working solution was added to each well and incubated for another 4 h. After removal of the medium, 200 μL DMSO was added to each well to dissolve the formed formazan. The plates were vortexed for 10 min to ensure complete dissolution. Then the OD was acquired with a microplate reader at 490 and 630 nm. The cell growth inhibition rate was calculated with the following equation: % inhibition = [1 – (sample group OD_490_ – sample group OD_630_)/(control group OD_490_ – control group OD_630_)] × 100%.

## Data Availability Statement

The raw data supporting the conclusions of this article will be made available by the authors, without undue reservation.

## Ethics Statement

The animal study was reviewed and approved by Weifang Medical University Ethics Committee.

## Author Contributions

LZ and WS designed the project. YC synthesized the molecules. JF performed the enzymatic screening and performed the *in vivo* antitumor experiment. YH and XW performed the *in vitro* antitumor experiments. YC and LZ analyzed the data and wrote the manuscript. All authors contributed to the article and approved the submitted version.

## Conflict of Interest

The authors declare that the research was conducted in the absence of any commercial or financial relationships that could be construed as a potential conflict of interest.
