# Supplementary material for: Discovery of N-(2-Amino-4-Fluorophenyl)-4-[bis-(2-Chloroethyl)-Amino]-Benzamide as a Potent HDAC3 Inhibitor
Source: Front Oncol. 2020 Oct 15;10:592385. doi: 10.3389/fonc.2020.592385 (PMC7593677; doi:10.3389/fonc.2020.592385)
Supplement: Supplementary file 1 [file Data_Sheet_1.PDF]

Spectra of FNA

H NMR spectra

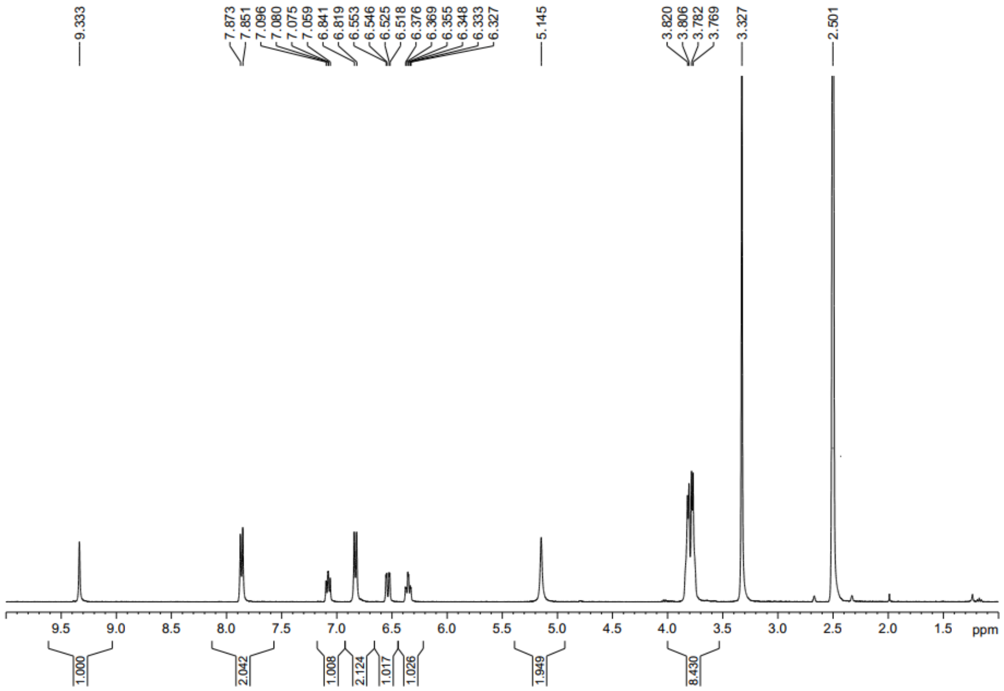

C NMR spectra

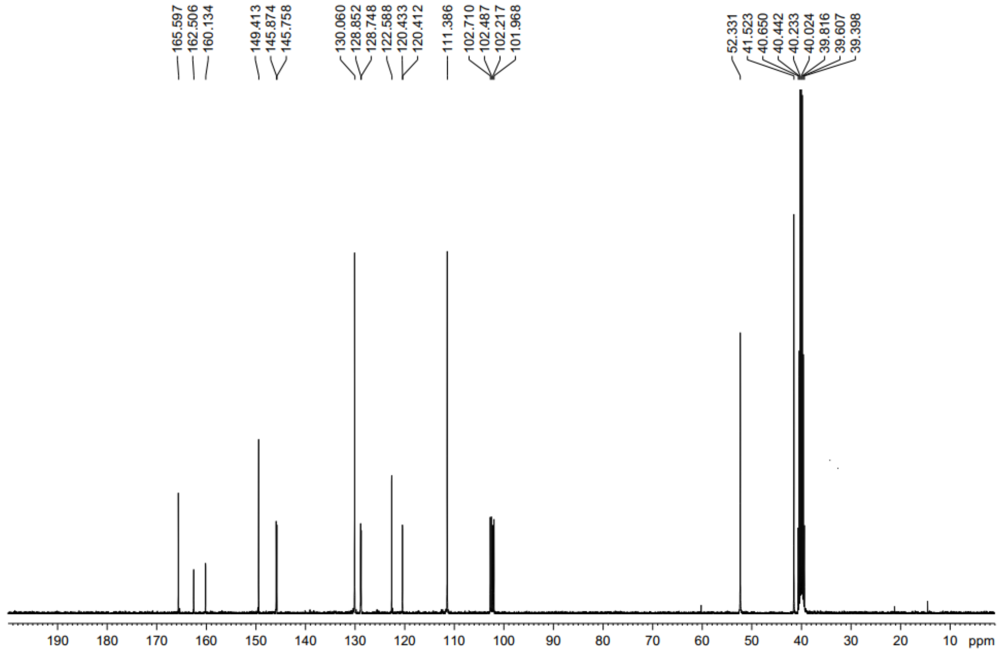

MS spectra

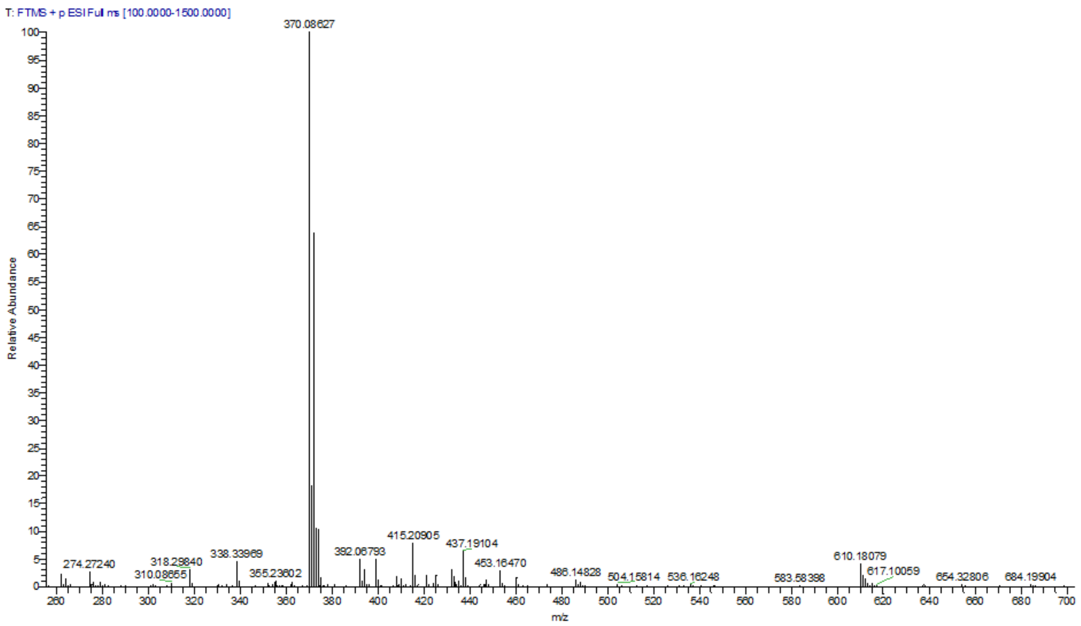

HPLC spectra

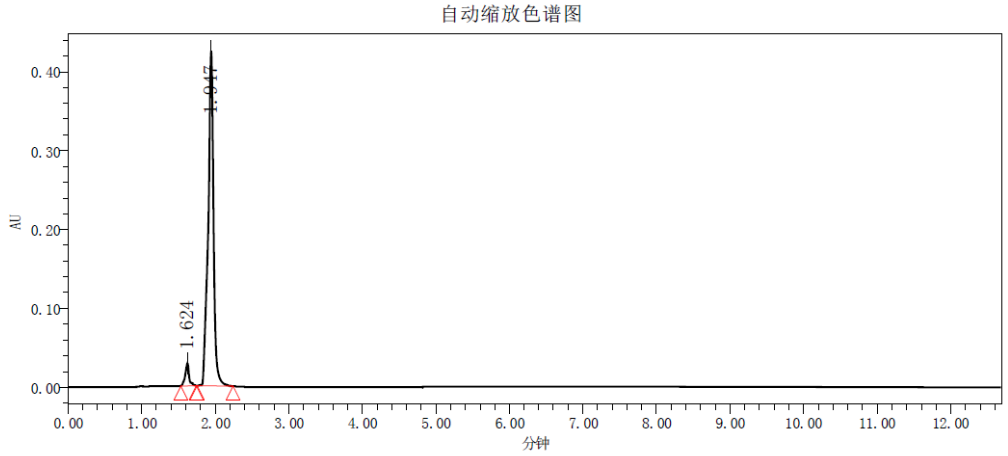

峰结果

	名称	保留时间(分钟)	面积(微伏*秒)	高度(微伏)	含量	单位
1		1.624	102782	28562		
2		1.947	2169198	424663		

处理通道说明: PDA Ch3 220 nm@4.8 nm

	处理通道说明	保留时间(分钟)	面积(微伏*秒)	% 面积	高度(微伏)
1	PDA Ch3 220 nm@4.8 nm	1.624	102782	4.52	28562
2	PDA Ch3 220 nm@4.8 nm	1.947	2169198	95.48	424663
